# Tetrandrine Combined with Gemcitabine and Cisplatin for Patients with Advanced Non-Small Cell Lung Cancer Improve Efficacy

**Published:** 2012-03

**Authors:** Wenchao Liu, Ju Zhang, Cheng Ying, Qianrong Wang, Chen Yan, Yang Jingyue, Yu Zhaocai, Xue Yan, Shi Heng-jun, Jiang Lin

**Affiliations:** 1 *Department of Oncology, Xijing Hospital, State Laboratory of Cancer Biology, the Fourth Military Medical University, Xi’an 710032, China;*; 2 *Institute of Gene Diagnosis, State Key Laboratory of Cancer Biology, Department of Pharmacology, the Fourth Military Medical University, Xi’an, 710032, China;*; 3 *Department of Traditional Medicine, Tangdu Hospital, the Fourth Military Medical University, Xi’an 710038, China*

**Keywords:** tetrandrine, non-small cell lung cancer, GP regimen, chemotherapy

## Abstract

Lung cancer has the highest morbidity and mortality of any malignant tumor. To improve efficacy and reduce toxicity in patients with advanced non-small cell lung cancer (NSCLC), it is important to integrate traditional and conventional medicine. Two hundred and forty patients with advanced NSCLC were randomized to tetrandrine plus GP or GP only. We infused gemcitabine on days 1 and 8; cisplatin on day 1. The tetrandrine group received continuous i.v. infusion for 10 days, with treatment repeated every 21 days. After 2 consecutive treatment cycles, we used RECIST criteria to evaluate short-term efficacy. Quality of life (QOL) was assessed according to Karnofsky score (KPS) and body weight change. We used NCI CTC 3.0 to evaluate treatment toxicity. The short-term objective response rate was 36.1% in the tetrandrine group and 24.3% in the controls (*P*=0.057). The short-term disease control rate was 63.9% in the tetrandrine group and 52.3% in the controls (*P*=0.081). The 1-year survival rates were 45.7% and 31.3%, respectively (*P*=0.059). KPS scores improved by 49.1% and 32.4%, respectively (*P*=0.012). Body weight increased by 28.7% in the tetrandrine group and 16.2% in the controls (*P*=0.027). The incidence of grade 2-4 leukopenia, thrombocytopenia, nausea, and vomiting in the tetrandrine group was 38.0%, 19.4%, 46.3%, and 16.7%, respectively; the control group figures were 53.2%, 34.2%, 63.0% and 27.9% (*P*<0.05). Tetrandrine may improve short-term efficacy and survival in patients with advanced NSCLC. Tetrandrine may also mitigate adverse reactions to chemotherapy and improve QOL for patients with NSCLC.

## INTRODUCTION

Lung cancer is the leading malignant tumor in terms of morbidity and mortality, with non-small cell lung cancer (NSCLC) accounting for approximately 80-85% of cases. Approximately 50% of patients are diagnosed too late for surgical treatment (1). Chemotherapy-based pharmacological treatment is the primary approach for the management of advanced NSCLC. Its goal is to increase survival time and improve quality of life (QOL) Chemotherapy is usually a platinum-based regimen that includes cisplatin or carboplatin combined with paclitaxel, docetaxel, gemcitabine, or vinorelbine (2). New chemotherapy agents are known to lower toxicity and increase efficacy and safety, but they are very expensive and offer limited advantages (3, 4). Thus, it is important to integrate traditional and conventional treatments to improve efficacy and reduce toxicity in patients with advanced NSCLC.

Tetrandrine (Tet) is known to be the main component of the alkaloid extracted from the root tuber of Stephaniatetrandra S. Moore which belongs to the plants of menispermaceae and Japanese stephania root. Tet with the chemical constitution of the class of bisbenzylisoquinoline, and the molecular formula of C_33_H_42_N_2_O_6_, is the main active ingredient of Stephaniatetrandra S. Moore. Up to date, the pharmacological studies indicate that Tet is a kind of calcium blocker, while it affects the transmembrane transport and the distribution and utilization of calcium ion inside cells by primarily blocking the calcium channel (5).

Recent years, several research groups have focused their effort on the effective constituent (Tet) of traditional Chinese drug that exhibits anticancer effect in various types of tumor cells (6, 7). Lee JH *et al* studied the molecular mechanism of growth inhibiting and apoptosis inducing when Tet treated pulmonary carcinosis cell line A 549. Then according to the cell-based cytotoxicity assay, they discovered that the toxicity of Tet to A 549 was time-dependent. The flow cytometry certified that Tet increased the number of cells in the cell cycle of second G1 and G1. The proliferation-inhibited effect of Tet associated with the decrease of the inhibiting factor of protein kinase of cell cycle dependence (CDK I, p21) and cyclin D1, and was also correlated with the path of apoptotic signals which included the activation of caspase-3, the destruction of cytoskeleton consisting of the distribution of F-actin, the increase of the separation of cell matrix, and the reduction of cellular microtubule protein α-tubulin, signifying that Tet, as one kind of cellular retarder and timorous chemopreventive agent, is worth being studied further (8).

With two different human bladder cancer cell lines, 5637 and T24, which represent high-risk superficial bladder cancer [5637] and high-grade bladder cancer (T24), Li X *et al* confirmed that Tet merits further *in vivo* investigation as a novel bladder cancer chemopreventive and chemotherapeutic agent in the clinical setting by testing Tet-induced apoptosis and growth inhibition (9). The research investigated by Meng *et al* indicated that Tet can obstruct the cellular generation in the cycle of G1 which resulted in the apoptosis of colon carcinoma of human called HT 29 (10). Some studies about the multidrug resistance (MDR) effect of Tet have been reported home and abroad (11, 12). Tet is akind of nonselective antagonist of calcium channel. Meanwhile, Tet is also the antagonist to calmodulin. Most of the researches suggested that Tet connected with P-gp causing the increase of the concentration of drugs accumulated inside tumor cells and the reversal of the drug resistance. Liu *et al* discovered that Tet had a more powerful MDR reversal effect on DNR, VLB, and DOX on the multidrug-resistant human T lymphoblastoid leukemia MOLT-4 cells which more highly express P-gp than the foregone P-gp inhibitor cyclosporin A, while Tet did not have such influence on the original MOLT-4 or MOLT-4 with low P-gp expression. In addition, the research found that both Tet and CsA could raise the accumulation of Rh123 inside the cells with drug resistance.

Studies indicate that the traditional Chinese patent medicine, tetrandrine, is effective against tumors and relieves some effects of treatment toxicity (13). However, no well-executed clinical trials have been conducted on the efficacy of chemotherapy combined with tetrandrine, or the treatment’s effect on QOL in patients with advanced NSCLC. In this paper, we investigated the effects of tetrandrine (Tet) injection combined with GP in patients with advanced NSCLC.

## MATERIAL AND METHODS

### Patients

NSCLC patients were enrolled from September 2007 to February 2009 in the Oncology Center, Xijing Hospital and Integrated Treatment Centre, Tangdu Hospital, the Fourth Military Medical University. We used the World Health Organization (WHO) tumor-node-metastasis (TNM) staging system and diagnosis by pathology to determine that all patients had stage IIIB-i.v. NSCLC. Karnofsky Performance Status (KPS) scores were >70. Patients were 18 to 75 years of age, and had at least one measurable lesion (CT-based longest diameter ≥10 mm). Antitumor treatment was stopped for all participants 1month prior to the start of the study. Each had an estimated survival time >12 weeks and normal function of major organs (e.g., heart, liver, kidneys). Patients receiving simultaneous treatment with other antitumor therapies and radiotherapy, and those with uncontrollable systemic diseases, allergic diathesis, or mental illness were excluded from the study, as were pregnant or breastfeeding women. The study protocol was approved by Institutional Review Board of the Ethics Committee of Xijing Hospital. All patients signed written informed consent forms. Patients who could not tolerate the toxicity were excluded from the efficacy analysis, but included in the safety analysis.

### Treatment

Gemcitabine hydrochloride (Gemzar®, Lilly France) 1000 mg/m^2^ in 0.9% sodium chloride injection (NS) 100 mL was administered on days 1 and 8 by i.v. infusion over 30 minutes. For the first 3 days, Cisplatin 75 mg/m^2^ in 500 mL NS was administered by i.v. infusion over 2 hours. Patients randomized to the treatment group also received tetrandrine (Yixian^®^, Jiangxi Yintao Pharmaceutical Co., Ltd. (Fuzhou, China) 150 mg in 500 mL NS via slow i.v. infusion for 10 days. Controls received the GP regimen only, with treatment repeated every 3 weeks. All patients had hematology, liver, and renal function tests each week during treatment. Efficacy and toxicity were evaluated after 2 consecutive treatment cycles. Treatment was continued for patients with no response or stable disease. The duration of treatment was 4-6 cycles. Patients with bone marrow suppression were supported by granulocyte colony-stimulating factor (G-CSF) to prepare for the next cycle of treatment.

### Outcome measures and evaluation criteria

The primary outcome measures were short-term objective response rate (RR), disease control rate (DCR), improvement in performance status score, incidence of adverse reactions, and median progression-free survival (mPFS). Secondary measures included median overall survival (mOS) and 1-year survival rate.

### Short-term objective efficacy

We evaluated efficacy according to Response Evaluation Criteria in Solid Tumors (RECIST) (14). Responses were classified as complete response (CR, all lesions resolved); partial response (PR, measurable lesions reduced by n Soli; stable or progressive disease (SD, measurable lesions reduced by <30% or increased by <20%); and progressive disease (PD, measurable lesions increased by ≥20% or development of new lesions). For patients evaluated as CR or PR, the response was confirmed by lesion measurement after at least 4 weeks. Short-term objective response rate was calculated as RR=CR+PR. Disease control rate was calculated as DCR=CR+PR+SD.

### Quality of life

We used the Karnofsky (KPS) score to evaluate performance status. A post-treatment increase in score of ≥10 was rated as an improvement; a decrease of ≥10 was rated as a decline; and a change of <10 points was rated as stable. An increase in body weight of >1.5 kg after treatment was rated as an increase; a decrease of >1.5 kg was rated as a reduction; body weight fluctuation within 1.5 kg was rated as stable (15).

### Adverse reactions

We used the National Cancer Institute Common Toxicity Criteria (NCI-CTC) 3.0 to evaluate adverse reactions (16).

### Survival data

PFS was defined as the period from enrollment to disease progression. Overall survival (OS) was defined as the time from the day when the patient received treatment until death or the last visit.

### Statistical analysis

We defined a 10% increase in relative risk as clinically significant. The SPSS 12.0 statistical software package (SPSS, Inc., Chicago, IL) was used to perform statistical analyses. Measurement data were compared by *t*-test. Enumeration data were compared by the χ^2^ test. P values less than 0.05 were considered significant.

## RESULTS

### Patients

We enrolled 240 NSCLCL patients from September 2007 to February 2009. They were randomly assigned according to a random number table to the tetrandrine group (tetrandrine + chemotherapy, n=120) or the control group (chemotherapy only, n=120). Of these patients, 16 refused the therapy and 5 required a change in therapy due to rapid disease progression. Baseline characteristics of the remaining 219 patients (108 in the tetrandrine group; 111 controls) are summarized in Table 1. There were no significant differences between the two groups in terms of gender, age, KPS score, pathological type of NSCLC, and clinical stage (*P*>0.05).

**Table 1:** Baseline characteristics of patients

	Tetrandrine group	Control group

n	108	111
Gender		
Male	81	72
Female	27	39
Age (years)		
≤40	11	18
41-59	41	44
≥60	56	49
Median age (years)	58.92	57.11
Karnofsky score		
70	18	18
80	45	42
90	45	51
Pathological type		
Adenocarcinoma	52	51
Squamous carcinoma	41	37
Adenosquamous carcinoma	4	7
Large cell carcinoma	11	15
Carcinosarcoma	0	1
Clinical stage		
IIIB	56	44
IV	52	67

### Short-term efficacy

The remaining 219 patients received at least 2 cycles of treatment, equivalent to 696 cycles of GP chemotherapy, including 341 cycles in the tetrandrine group, and 355 in the control group. The mean dose intensity was similar between the two groups.

After 2 consecutive treatment cycles, 5 patients in the tetrandrine group (n=108) had a CR; 34 had a PR; 30 had SD; and 39 had PD. RR was 36.1% and DCR was 63.9%. After 2 consecutive treatment cycles, 2 control group (n=111) patients had a CR; 25 had a PR; 31 had SD; and 53 had PD. RR was 24.3% and DCR was 52.3%. The χ^2^ test indicated no significant differences between the groups for RR (*P*=0.057) and DCR (*P*=0.081) (Table 2).

**Table 2:** Short-term efficacy by patient group

Group	n	Efficacy				RR=CR+PR (%)	DCR=CR+PR+SD (%)
		CR	PR	SD	PD		

Tetrandrine	108	5	34	30	39	39 (36.1)	69 (63.9)
Control	111	2	25	31	53	27 (24.3)	58 (52.3)

### Performance status

After treatment, KPS scores increased in 53 patients, was stable in 45, and decreased in 10 patients in the tetrandrine group. The corresponding numbers in the control group were 36, 56, and 19. After treatment, a KPS score increase ≥10 was seen in 49.1% of the patients in the tetrandrine group and 32.4% of those in the control group (*P*=0.012) (Table 3).

**Table 3:** Change in Karnofsky score after treatment

Group	n	Increase	Stable	Decrease	Patients with score increase (%)

Tetrandrine	108	53	45	10	49.1^a^
Control	111	36	56	19	32.4

a *P*<0.05 versus control group.

### Body weight change

In the tetrandrine group, 31 patients gained weight, 59 remained stable, and 18 lost weight. The corresponding figures in the control group were 18, 63, and 30. The body weight of approximately 28.7% of the patients in the tetrandrine group increased by >1.5 kg after treatment; the proportion in the control group was 16.2% (*P*=0.027) (Table 4).

**Table 4:** Change in body weight (BW) after treatment

Group	n	Increase	Stable	Decrease	Patients with BW increase (%)

Tetrandrine	108	31	59	18	28.7^a^
Control	111	18	63	30	16.2

a *P*<0.05 versus control group.

### Adverse reactions

The main toxicities in both groups were bone marrow depression, liver impairment, gastrointestinal reaction, rash and itching, local reaction (pain), and alopecia. The incidence of hash and itching, local thrombocytopenia, nausea, and vomiting in the tetrandrine and the control groups was 38.0% *vs*. 53.2%, 19.4% *vs*. 34.2%, 46.3% *vs*. 63.0%, and 16.7% *vs*. 27.9%, respectively (*P*<0.05) (Table 5). The incidence of Grade 2-4 decreased hemoglobin (HB), abnormal liver or renal function, diarrhea, constipation, itching skin, local reaction (pain), and alopecia was not significantly different between the groups (Table 5). The incidence of Grade 1 local reaction in the tetrandrine group was higher than in the control group. No phlebitis was reported. Cold compress or wet dressing with magnesium sulfate was used to relieve local pain. No apparent fever or hypersensitivity reaction occurred in the tetrandrine group.

**Table 5:** Adverse reaction profiles after treatment (number of cases)

Adverse reaction	Tetrandrine group					Incidence of Grade 2 to 4 toxicity (%)	Control group					Incidence of Grade 2 to 4 toxicity (%)
	Grade 0	Grade 1	Grade 2	Grade 3	Grade 4		Grade 0	Grade 1	Grade 2	Grade 3	Grade 4	

WBC↓	36	31	34	6	1	38.0^a^	19	33	45	9	5	53.2
HB↓	77	16	13	2	0	13.9	69	21	16	5	0	18.9
Platelet↓	62	25	14	5	0	19.4^a^	46	27	27	9	2	34.2
Liver impairment	81	20	5	2	0	6.5	88	16	7	0	0	6.3
Renal impairment	95	13	0	0	0	0.0	94	17	0	0	0	0.0
Nausea	58	—	37	13	—	46.3^a^	41	—	47	23	—	63.0
Vomiting	55	35	17	1	0	16.7^a^	42	38	25	6	0	27.9
Diarrhea	94	14	0	0	0	0.0	90	21	0	0	0	0.0
Constipation	86	17	5	0	0	4.6	80	23	7	1	0	7.2
Rash and itching	93	15	0	0	—	0.0	93	16	2	0	—	1.8
Local irritation	77	28	3	0	0	2.8	99	12	0	0	0	0.0
Alopecia	74	24	10	—	—	9.3	67	31	13	—	—	11.7

a *P*<0.05 versus control group; “—”, means no cases.

### Survival

Follow-up on all 219 patients lasted until August 31, 2009. We obtained OS data from 156 of them, and FS data from 172. In the tetrandrine group, the median PFS was 172 days; the median survival period was 381 days. In the control group, the respective results were 151 and 338 days. The patients enrolled before August 31, 2008 (n=164) were included in the analysis on an intent-to-treat basis: the 1-year survival rate of the tetrandrine group (n=81) was 45.7%; the 1-year survival rate of the control group (n=83) was 31.3% (*P*=0.059) (Table 6).

**Table 6:** One-year survival rate of patients enrolled before August 31, 2008

Group	n	Survived after 1 year (n)	1 year survival rate (%)	*P* value

Tetrandrine	81	37	45.7	0.059
Control	83	26	31.3	

## DISCUSSION

Lung cancer is currently one of the most common malignant tumors in the world. According to the third survey report of national causes of death issued by the Chinese Ministry of Health, lung cancer accounts for 22.7% of all cancer deaths. The incidence of lung cancer is increasing. Approximately 80-85% of all lung cancer cases are NSCLC. Most NSCLC patients are diagnosed at an advanced stage in which radical surgery is no longer an option (17, 18). Chemotherapy is the mainstay therapy for advanced NSCLC.

The Eastern Cooperative Oncology Group (ECOG) Study 1594 and other multicenter clinical trials have established the roles of 4 third-generation chemotherapy regimens in the treatment of advanced NSCLC, as well as third-generation agents (gemcitabine, vinorelbine, paclitaxel and docetaxel) in combination with platinum agents. Both are recommended in clinical oncology guidelines developed within the past 6 years. The efficacy rate of these regimens ranges from 30% to 55% (19). However, high toxicity of chemotherapy results in lower QOL (20, 21).

Gemcitabine plus cisplatin is one of the most effective regimens for NSCLC, and cisplatin is one of the most important therapeutic agents. Platinum-based chemotherapy regimens can significantly improve survival time and the 1 year survival rate of NSCLC patients (22). Cell-cycle specific antimetabolites in Gemzar (gemcitabine hydrochloride) mainly target tumor cells at the DNA synthesis phase. Under certain conditions, these agents can prevent the progression of the cell cycle from the G1 phase to the S phase. Gemcitabine, as a prodrug, is a good substrate of deoxythymidine kinase phosphorylation in cells. It is metabolized to active nucleoside diphosphate (dFdCDP) and nucleoside triphosphate (dFdCTP) by nucleoside kinase. Its cytotoxic activity lies in the combined inhibitory effect of these two nucleosides on DNA synthesis.

Combined with cisplatin, gemcitabine produces synergistic and additive antitumor effects and improves clinical efficacy (22-24). However, combination chemotherapy usually leads to varying degrees of toxicity and multidrug resistance (19). Therefore, research on lung cancer chemotherapy is currently focused on how to improve efficacy, ameliorate clinical symptoms, and alleviate adverse effects.

Tetrandrine is a bisbenzylisoquinoline alkaloid extracted from the dried root tuber of *Stephania tetrandra*, a *Menispermaceae* plant (23). Early experiments have shown that it has extensive pharmacological effects (e.g., antihypertensive, anti-inflammatory, antifibrotic, analgesic, toxicity-reducing, and anti-tumor actions). Its effect on renal function is minimal, as are other adverse effects. In clinical settings, tetrandrine has been effectively used to treat a variety of diseases (e.g., hypertension, silicosis, liver fibrosis) (24, 25). Clinical reports on the medication’s anti-tumor activity have focused primarily on radiotherapy sensitizing or reversal of multidrug resistance (MDR) in hematological malignancies. Larger studies are needed to determine its effect as an anti-tumor agent (26).

Many experiments have demonstrated that tetrandrine has a number of anti-tumor mechanisms. It can inhibit the growth of tumor cells by inducing apoptosis, sensitizing radiotherapy, and protecting against potentially lethal injury caused by radiation. It can also alleviate radiation damage by inhibiting inflammatory reactions. Tetrandrine can reduce the toxicity of chemotherapy by relieving alveolar inflammation before the formation of lung fibrosis induced by peplomycin and bleomycin, expedite the removal of oxygen radicals, and avoid release of various profibrotic factors due to damage of functional lung cells.

It can also reverse MDR primarily associated with amplification of the intracellular multidrug resistance gene (mdr) and overexpression of P-glycoprotein (P-gp). P-gp is an energy-dependent drug pump that can drain many anti-tumor drugs out of cells, reducing intracellular drug accumulation. Thus, cells show the phenotype of MDR. Synergy between tetrandrine and chemotherapy can indirectly block the efflux pump function of membrane-bound surface P-gp. This reverses drug resistance by increasing the intracellular level of chemotherapy agents (27, 28).

In this study, KPS score and body weight outcomes suggest that tetrandrine can improve QOL. Fewer adverse reactions were also observed in the tetrandrine treatment group compared with the control group, including alleviation of chemotherapy-induced Grade 2-4 nausea and vomiting, and prevention of Grade 2-4 leucopenia and thrombocytopenia. The latter may be associated with toxicity-reducing and immunity-enhancing effects of tetrandrine. Local infusion site irritation was greater in the tetrandrine group than in the control group, but no phlebitis was reported. Cold compresses or wet dressings with magnesium sulfate relieved local pain. No apparent fever or hypersensitivity reaction occurred in the tetrandrine group, and adverse effects were tolerable and able to be addressed by reducing the concentration of the tetrandrine infusion or using a local preventive wet dressing (29).

A RR and one1-year survival rate increase of 10% is defined as clinically significant efficacy. According to the formula used to calculate the sample size in clinical trials that compare these two rates, at least 198 patients per group are required, or a total of 396 patients. A limitation of this study is its small sample size. Nonetheless, the results show that tetrandrine injection plus GP chemotherapy may improve short-term efficacy and increase survival in advanced NSCLC compared with the GP only regimen. Short-term response, clinical benefit, and 1 year survival in the tetrandrine plus GP chemotherapy group increased significantly compared with the GP-treated group. These findings may have clinical implications. However, large multicenter, randomized, controlled trials are needed to confirm our outcomes.

Our data suggest that tetrandrine combined with GP can improve the short-term efficacy of chemotherapy and increase survival in patients with advanced NSCLC. This regimen did not show any significant toxicity, and it also alleviated chemotherapy-induced adverse reactions (e.g., bone marrow depression, nausea, vomiting). The regimen appears able to improve QOL in patients with advanced NSCLC, and is worthy of expanded use in clinical practice and further clinical study.

## RANDOMISED CONTROLLED TRIALS

In this randomized controlled trial, Tetrandrine in combination with gemcitabine + cisplatin improved the short-term efficacy and survival in patients with advanced NSCLC as compared with gemcitabine + cisplatin alone.

Tetrandrine may reduce the incidence of adverse reactions associated with gemcitabine+cisplatin chemotherapy and improve the quality of life for patients with NSCLC.
